# Suicide by ingestion of caffeine

**DOI:** 10.1186/s41935-017-0001-2

**Published:** 2017-07-19

**Authors:** Frédéric Aknouche, Emilie Guibert, Alison Tessier, Aude Eibel, Pascal Kintz

**Affiliations:** 1 Laboratoire Aknouche, 22 avenue Robert Soleau, 06000 Antibes, France; 2 Institut de Médecine légale, 11 rue Humann, 67000 Strasbourg, France; 3 X-Pertise Consulting, 84 route de Saverne, 67205 Oberhausbergen, France

**Keywords:** Caffeine, suicide, blood, post-mortem, toxicology

## Abstract

**Background:**

Mr K, aged 48, was found sweating by his partner at home at 11.50 pm. He claimed to have attempted suicide. She immediately called the Emergency Unit to ask for support. At the phone, the physician on duty indicated her to give a pill of Lysanxia (prazepam) to decrease the level of stress of the victim. However, the clinical situation worsened and he was taken to the hospital at 1.00 am. At his arrival at 1.28 am he was in cardiac arrest. Despite intensive resuscitation manoeuvres, death was pronounced at 2.30 am. At home, an empty plastic bag with a 100 g caffeine label was found. The drug was bought via Internet 6 months earlier. External body examination and autopsy revealed the lack of any traumatic injury.

**Findings:**

During examination, the pathologist collected peripheral blood (femoral blood). This specimen was tested for ethanol, volatiles, pharmaceuticals and drugs of abuse, using head space GC/FID and GC/MS, ELISA, LC-DAD, GC/MS and LC/MS/MS. Ethanol tested negative in blood. Using a dedicated LC/MS/MS procedure, caffeine was identified at 401 mg/l, which can correspond to a fatal concentration. Nordiazepam, sertraline and fluoxetine, the prescribed medications of the victim, were identified at therapeutic concentrations, 188, 31, and 48 ng/ml, respectively. Amiodarone was also identified at high concentration (4200 ng/ml), part of the medical assistance of the rescue team.

**Conclusion:**

The manner of death was considered as acute intoxication with caffeine.

## Introduction

Caffeine or 1,3,7-trimethylxanthine is a basic alkaloid that occurs naturally in many plants distributed world-wide, including cocoa and coffee beans, kola nuts, guarana berries, yaupon holly leaves and tea leaves. The total caffeine amount in these materials can be up to 2% by weight. Extraction of caffeine from beans, nuts or leaves can be performed using various organic solvents, such as benzene or chloroform. However, due to environmental and health issues, caffeine is more and more prepared after water extraction or supercritical carbon dioxide extraction.

Caffeine is a central nervous system stimulant and is consumed to reduce physical fatigue, by delaying or preventing sleep. The drug is used to improve task performance. It also produces respiratory and myocardial stimulation, diuresis and coronary vessel dilatation. The amount of caffeine needed to produce these effects varies from person to person, depending on body size and degree of tolerance. Effects begin about 1 h after administration and the drug is active for 3 to 4 h.

Only a few fatal caffeine intoxication cases have been reported in the medical literature. Death due to caffeine overdose is rare, due in part to its marked gastric irritation resulting in spontaneous emesis. Most of these cases are suicides and a recent review (Silva et al. 2014) has demonstrated that, despite being an addictive substance and potentially fatal in higher doses, caffeine was still a rare factor in a number of studies concerning its relation with suicide attempts and death.

Caffeine is one of the drugs most commonly used by the public, irrespective of the countries. It has long been recognized as an addictive substance and numerous toxic effects, including vomiting, abdominal pain, agitation, altered conscious state, supraventricular and ventricular tachyarrythmias, and seizures have been described. Death due to caffeine overdose is considered rare and mostly under suicidal circumstances. The direct cause of death is often described to be ventricular fibrillation.

An average cup of coffee contains about 80 to 100 mg of caffeine, resulting in a plasma concentration up to 2 mg/l. Numerous dietary supplements also contain caffeine with doses ranging from 30 to 200 mg, sometimes even higher, leading to a concentration up to 10 mg/l. In order to achieve a fatal concentration, a subject would need to ingest 5 to 10 g of pure caffeine (Yamamoto et al. 2015).

## Case report

Mr K, aged 48 and measuring 1.75 m, was found sweating by his partner at home at 11.50 pm. He claimed to have attempted suicide by ingestion of 100 g of caffeine. She immediately called the Emergency Unit to ask for support. At the phone, the physician on duty indicated her to give a pill of Lysanxia (prazepam) to decrease the level of stress of the victim. However, the clinical situation worsened and he was taken to the hospital at 1.00 am. At his arrival at 1.28 am he was in cardiac arrest with marked cyanosis. Despite intensive resuscitation manoeuvres (cardiac massage, electric impulsions, intubation, administration of vasoactive drugs), death was pronounced at 2.30 am.

At home, an empty plastic bag with a 100 g caffeine label was found. The drug was bought via Internet 6 months earlier. The day before the suicide, the subject was surfing on the Internet to get information about the toxicity of caffeine. The man wanted to commit suicide by hanging 13 years before this attempt.

External body examination and autopsy revealed generalized organ congestion and the lack of any traumatic injury (confirmed by radiology). The subject was in overweight status. Cyanosis of both arms and legs was noticed. Cyanosis of nails was also present. A mixture of powder and food was identified in the stomach content. During examination, the pathologist collected femoral blood.

There was a delay in transferring the patient to the hospital, and earlier referral might have saved his life. At the time of writing, the case is still under investigation by the prosecutor.

## Toxicological analyses

Ethanol was tested by head space GC/FID on a Perkin Elmer system (TurboMatrix 40 & Clarus 580) using a standard validated procedure. Volatiles were tested by head space GC/MS on a Thermo system (Focus GC & DSQII) using a standard validated procedure. ELISA tests were achieved using Concateno Cozart Microplate kits using the recommendations of the manufacturer. Given the circumstances of death, caffeine and other pharmaceuticals were tested by screening methods involving GC-MS, LC-DAD and LC-MS/MS.

Briefly, caffeine was extracted from 0.1 ml in presence of 200 ng of prazepam-d_5_ used as internal standard by 0.1 ml saturated ammonium chloride pH 9.5 buffer (adjusted with ammonia water) and 0.8 ml of dichloromethane/*n*-heptane/isopropanol (25/65/10, v/v). After extraction, centrifugation and evaporation to dryness, the residue was reconstituted in 50 μl of mobile phase. LC was performed using a Waters Xevo TQD system. Chromatography was achieved using a Waters HSS C18 column (150 × 2.1 mm, 1.8 μm).

In blood, linearity was observed for caffeine concentrations ranging from 10 to 500 mg/l with a correlation coefficient of 0.999. Within-batch precision at 50 mg/l was 12.1%. The limit of detection was estimated to be 0.01 mg/l, with a S/N ratio of 3. Under the chromatographic conditions used, there was no interference with the analytes by chemicals or any extractable endogenous materials present in blood (matrix effect < 20%).

## Results and discussion

Ethanol tested negative in blood. ELISA screening was positive for benzodiazepines. HbCO was 1.5% and cyanides were at physiological concentrations (<80 ng/ml). No drug of abuse was detected. Using a LC/MS/MS procedure, caffeine was identified in the femoral blood at 401 mg/l, which can correspond to a fatal concentration. Nordiazepam, sertraline and fluoxetine, the prescribed medications of the victim, were identified at therapeutic concentrations, 188, 31, and 48 ng/ml, respectively. Amiodarone was also identified at high concentration (4200 ng/ml), part of the medical assistance of the rescue team. This cannot be considered to account for the final outcome. There was no attempt to test for the metabolites (paraxanthine, theobromine, 1, 3 or 7-methylxanthine), although they were observed during the screening process.

The fatal blood concentration is in the range 80-100 mg/l (Yamamoto et al. 2015). As a consequence, the measured concentration in the present case can be listed as potentially lethal.

Since several years, some case reports have been published (Yamamoto et al. 2015; Winek et al. 1985; Mrvos et al. 1989; Riesselmann et al. 1999; Holmgren et al. 2004; Kerrigan & Lindsey 2005; Jabbar & Hanly 2013; Banerjee et al. 2014; Bonsignore et al. 2014). Blood concentrations reported in the literature are presented in Table 1. As caffeine may exhibit post-mortem redistribution (heart to femoral blood ratio of 1.2 in average), it is important to document the anatomical site of blood collection (Riesselmann et al. 1999; Dalpe-Scott et al. 1995). Plasma to blood ratios of 0.93 have been reported (Kerrigan & Lindsey 2005).

**Table 1:** Reported fatal caffeine concentrations in the literature

Reference	Number of cases	Type of blood	Caffeine concentration (mg/l)
this case	1	femoral	401
2	1	femoral	290
3	1	unknown	240
4	1	serum	1560
5	2	femoral	190 – 220
6	4	femoral	153 – 173 – 200 – 210
7	2	femoral	192 – 567
8	1	unknown	350
9	8	unknown	average at 140.4
10	1	femoral	170

One should also take into consideration that the response and tolerance to caffeine are individual and depend on pharmacokinetics and pharmacodynamics. In addition, genetic variability in caffeine-metabolizing enzymes affects the susceptibility of each individual to caffeine toxicity.

Based on the absence of pathology on autopsy and the high caffeine level in blood, death was attributed to acute caffeine toxicity. As it is easy to detect the drug during standard toxicological screening, the authors recommend to quantify caffeine concentrations in all cases in order to include/exclude it as a cause of death.

## Conclusion

Despite the low incidence of caffeine toxicity, the availability of caffeine-containing drugs, including over-the-counter stimulant products and energy drinks, raises serious questions regarding the possible risk of caffeine overdose. Increasingly being sold as a dietary supplement, many people, particularly those in the health and fitness community, where it is touted as a fitness and building aid, are consuming anhydrous caffeine on a regular basis.

This case illustrates that deaths resulting from caffeine, although rare, continue to occur. It emphasizes the usefulness of performing exhaustive toxicology and searching for all potentially relevant information in order to formulate appropriate hypotheses concerning the cause and manner of death. A very recent review about caffeine fatalities has been published recently (Jones 2017 Feb), which confirms our findings.
